# Stratification of a Phelan–McDermid Syndrome Population Based on Their Response to Human Growth Hormone and Insulin-like Growth Factor

**DOI:** 10.3390/genes14020490

**Published:** 2023-02-15

**Authors:** Bridgette A. Moffitt, Sara M. Sarasua, Diana Ivankovic, Linda D. Ward, Kathleen Valentine, William E. Bennett, Curtis Rogers, Katy Phelan, Luigi Boccuto

**Affiliations:** 1 School of Nursing, Healthcare Genetics Program, Clemson University, Clemson, SC 29634, USA; 2 Division of Pediatric Gastroenterology, Hepatology, and Nutrition, Indiana University, Riley Hospital for Children, Indianapolis, IN 46202, USA; 3 Greenwood Genetic Center, Greenwood, SC 29646, USA; 4 Genetics Laboratory, Florida Cancer Specialists & Research Institute, Fort Myers, FL 33916, USA

**Keywords:** Phelan–McDermid syndrome, PMS, 22q13.3 deletion syndrome, *SHANK3*, growth hormone, hGH, insulin-like growth factor 1, IGF-1

## Abstract

Phelan–McDermid syndrome (PMS), caused by pathogenic variants in the *SHANK3* gene or 22q13 deletions, is characterized by intellectual disability, autistic features, developmental delays, and neonatal hypotonia. Insulin-like growth factor 1 (IGF-1) and human growth hormone (hGH) have been shown to reverse neurobehavioral deficits in PMS. We assessed the metabolic profiling of 48 individuals with PMS and 50 controls and determined subpopulations by taking the top and bottom 25% of responders to hGH and IGF-1. A distinct metabolic profile for individuals with PMS showed a reduced ability to metabolize major energy sources and a higher metabolism of alternative energy sources. The analysis of the metabolic response to hGH or IGF-1 highlighted a major overlap between both high and low responders, validating the model and suggesting that the two growth factors share many target pathways. When we investigated the effect of hGH and IGF-1 on the metabolism of glucose, the correlation between the high-responder subgroups showed less similarity, whereas the low-responders were still relatively similar. Classification of individuals with PMS into subgroups based on responses to a compound can allow an investigation into pathogenic mechanisms, the identification of molecular biomarkers, an exploration of in vitro responses to candidate drugs, and eventually the selection of better candidates for clinical trials.

## 1. Introduction

Phelan–McDermid syndrome (PMS), also known as 22q13.3 deletion syndrome (OMIM #606232), is a rare neurodevelopmental disorder that can be caused by a terminal deletion or other rearrangement affecting the distal region of chromosome 22q, or pathogenic variants within the *SHANK3* gene [1,2]. *SHANK3* codes for a key scaffolding protein in the post-synaptic density playing an important role in the function and maintenance of excitatory synapses [3,4,5,6]. PMS can be characterized by developmental delays, mild to severe intellectual disability (ID), seizures, sleep disturbances, autism spectrum disorder (ASD), hypotonia, and other features [7,8]. Establishing a clinical diagnosis can be challenging because of the extreme variability in phenotype, and therefore confirmatory genetic tests are required [1,9].

The *SHANK3* gene encodes for a critical scaffolding protein of the post-synaptic density playing an essential role in the function and maintenance of excitatory synapses that are highly expressed neurons of the central nervous system [3,4,6,10]. According to Bozadgi et al. (2010), in a normally functioning postsynaptic site there are around 300 Shank molecules [6]. When Shank3 expression is inhibited, there is a reduction in the dendritic spines on the cultured neurons, and when Shank3 is reintroduced, these neurons develop more spines with functional synapses [6,11]. The spines located on the dendrite of a neuron are required to be functional to receive signals from the axon and transmit signals to the cell body of the neuron [11,12]. Recent studies have shown a significant reduction in Shank3 protein in brain tissues obtained from PMS patients [10]. In vivo studies demonstrated that *SHANK3* haploinsufficiency leads to a reduction in AMPA receptors and proper synaptic development [6,13,14]. In vivo studies in *Shank3*-KO mice showed severe behavioral abnormalities that are associated with ASD and related neurodevelopmental disorders, such as abnormal social behavior, impaired motor coordination, and repetitive self-injurious grooming [15,16,17]. Therefore, the loss or reduction in *SHANK3* can be correlated with poorly functioning neurons within the human body and displaying ASD-like behaviors. In vivo *Shank3*-deficit PMS models treated with insulin-like growth factor 1 (IGF-1) showed enhanced motor performance and functional improvements [13]. A *Shank3* mouse model showed behavioral improvements, reduced neuroinflammation, and increased *Igf1* levels when Hyperbaric Oxygen Therapy (HBOT) was administered [17].

Recently, several studies have explored using IGF-1 and human growth hormone (hGH) as therapeutic options for the treatment of PMS [18,19]. Insulin-like growth factor 1 aids in the development of mature synapses [20]. Many cell models have used commercially available IGF-1 to explore the potential of promoting the growth and branching of synapses to overcome neurodevelopmental disorders and neurodegenerative diseases [20,21]. Preliminary data from the first IGF-1 clinical trial reported behavioral and social improvements in nine individuals with PMS [3]. However, IGF-1 is costly and in short supply [18]. Further, due to the direct influence of IGF-1 on glucose metabolism, individuals receiving IGF-1 must be closely monitored for hypoglycemia [18,19]. To minimize costs and side effects, the less expensive hGH has been suggested as an alternative to IGF-1 treatment because it is capable of increasing IGF-1 levels without the risk of a hypoglycemic event [18]. A recent case study reported an individual with PMS treated with hGH who experienced improvements in motor skills and social behaviors without adverse side effects [22]. Targeted treatment of PMS is in the early stages, with only six published studies using intranasal insulin, IGF-1, and hGH [2,3,18,19,23]. However, many clinical trials that have aimed at treating neurodevelopmental disorders have encountered clinical and molecular inconsistencies that have hindered their success [20]. A method to identify patients most likely to benefit from IGF-1 or hGH is needed.

Therefore, the purpose of this study was to develop a test that may be used as an indicator of a good candidate for IGF-1 or hGH therapy. Our study aimed to (1) identify the metabolic profile of individuals with PMS, (2) specifically analyze the metabolic response of cells from these individuals to hGH and IGF-1, possibly identifying biomarkers related to such responses, and (3) stratify the PMS population into low and high responders for future use at determining good candidates for clinical trials.

## 2. Methods

### 2.1. Participants

Forty-eight individuals with confirmed PMS and fifty age-matched controls participated in this study. Participants were recruited at the Greenwood Genetic Center (GGC), Greenwood, South Carolina, and/or through the Phelan–McDermid Syndrome Foundation (PSMF) [24]. Parents or guardians of individuals provided written informed consent. The research was approved by the Self Regional Health System Institutional Review Board as a part of the project “Characterization of the relationship between genotype and neurobehavioral phenotype in individuals with Phelan-McDermid syndrome” IRB protocol number 00057738. The participants included 26 females (54.2%) and 22 males (45.8%), ranging in age from 3 years to 45 years old. The average age was 13.98 years old. Thirty-one individuals (64.6%) had deletions on 22q13 ranging from 0.05 megabases (Mb) to 8.45 Mb, with the average deletions size being 3.863 Mb. There were 9 individuals (18.8%) with a *SHANK3* pathogenic variant, and 8 individuals (16.6%) had deletions of unknown size.

### 2.2. Lymphoblastoid Cell Lines (LCLs)

Lymphoblastoid cell lines (LCLs) were established from lymphocytes collected from peripheral blood samples and immortalized via transfection with the Epstein–Barr virus. LCLs were harvested in Sigma RPMI-1640 with 15% fetal bovine serum (FBS), 2 mM L-Glutamine, 100 U/mL Penicillin, and 100 µg/mL Streptomycin [25]. The cells were selected for the experiments only if they showed a viability of 55% or higher.

### 2.3. Metabolic Profiling via Biolog Phenotype Mammalian Microarrays (PM-Ms)

To identify metabolic signatures of individuals with PMS, their cell lines were compared with 50 control LCLs, using specialized technology developed by Biolog (Hayward, California, USA) called Phenotype Mammalian MicroArray (PM–M) plates. The PM–M plates are designed to assess the metabolic activity by measuring the cellular production of NADH (nicotinamide adenine dinucleotide, reduced form) in the presence of different compounds. Each microarray plate contains diverse molecules, which either act as an energy source (plates PM–M1 to M4) or as metabolic effectors (plates PM–M5 to M8). Included on plate PM7 are 6 wells of varying concentrations of hGH and 6 wells of varying concentrations of IGF-1. A custom tryptophan plate (TRP) was developed by Biolog for GGC that used previously published data showing the use of tryptophan as an energy source decreased in individuals with ASD [25]. More in-depth methodology of these PM-M and TRP microarray plates and raw data have been published previously [26]. The relative absorbance (A_590-750_) was calculated per well and these absorbance endpoint readings were used as a measure of metabolite usage. Readings were normalized using triplicate absorbance readings from the corresponding empty plate (plates run with just media and dye, without cells). Data were analyzed using a custom R package (version 3.6) and the opm R package (available at R-Forge.r-project.org). These values were compared to the average values generated by 50 control individuals, which have been previously reported [27]. The goal was to identify significant abnormalities in metabolic pathways and response to effectors. To analyze the metabolic profiles, the non-parametric Mann–Whitney two-sided test was used to calculate P-values. To control for multiple testing, the Benjamini–Hochberg correction (R method: p.adjust) was applied with a false discovery rate (FDR) set to q < 0.05. This approach allowed the ability to identify compounds differentially metabolized between individuals with PMS and controls.

### 2.4. Selection of High and Low Responders

To determine high and low responders to hGH within our PMS cohort, the average absorbance of the six wells containing hGH on plate PM–M7 for the 48 individuals with PMS was taken. The top 25% and bottom 25% absorbance values were used to stratify the PMS population into two subpopulations based on the response to hGH. The same selection process was completed using the six wells of IGF-1 on plate PM–M7 for the same individuals within the PMS cohort. The top 25% and bottom 25% absorbance values were used to stratify the PMS population into two different subpopulations based only on their response to IGF-1. Once the four subpopulations were determined, those populations’ metabolic profiles were compared to the control group of 50 individuals across all PM–M plates (PM–M1 to M8, and TRP).

### 2.5. Correlation to Account for Baseline Cellular Response

Considering the risk of hypoglycemia described in association with IGF-1, to further explore the effect of hGH and IGF-1 on glucose metabolism, a correlation was conducted using the first six wells on plate PM–M7 (A1 to A6), which contained no metabolic effector. We assessed the utilization of the glucose in the media to produce NADH without any external influence and considered these six wells a control for the baseline metabolism of glucose. The average of these six control wells was taken and each well that contained hGH and IGF-1 (E1 to E12) for all 48 individuals with PMS was divided by those averages. For each of the hormones, we calculated a new relative average accounting for the correlation to the baseline glucose values. The top 25% and bottom 25% absorbance values for hGH and IGF-1 were used to identify two new subpopulations for responders that were based specifically on the metabolic effect of each of the hormones on glucose metabolism.

## 3. Results

### 3.1. Metabolic Profile of Individuals with PMS

The striking finding was the number of metabolites differentially utilized between the PMS cohort and the control cohort. The PMS LCLs showed an overall trend toward a lower production of NADH throughout all PM–M and TRP plates (Table 1). There was a significant reduction in the utilization of storage forms of energy that were related to glucose, such as glycogen and dextrin. Wells containing alternative energy sources showed a higher level of NADH production when compared to controls. The PMS cohort showed 210 out of a total of 776 wells with NADH levels significantly different from the control group. Of these 210 wells, 51 generated higher levels of NADH than controls and 159 wells generated lower levels. Six out of the eight wells on the TRP plate produced lower levels of NADH than controls.

**Figure 1:**
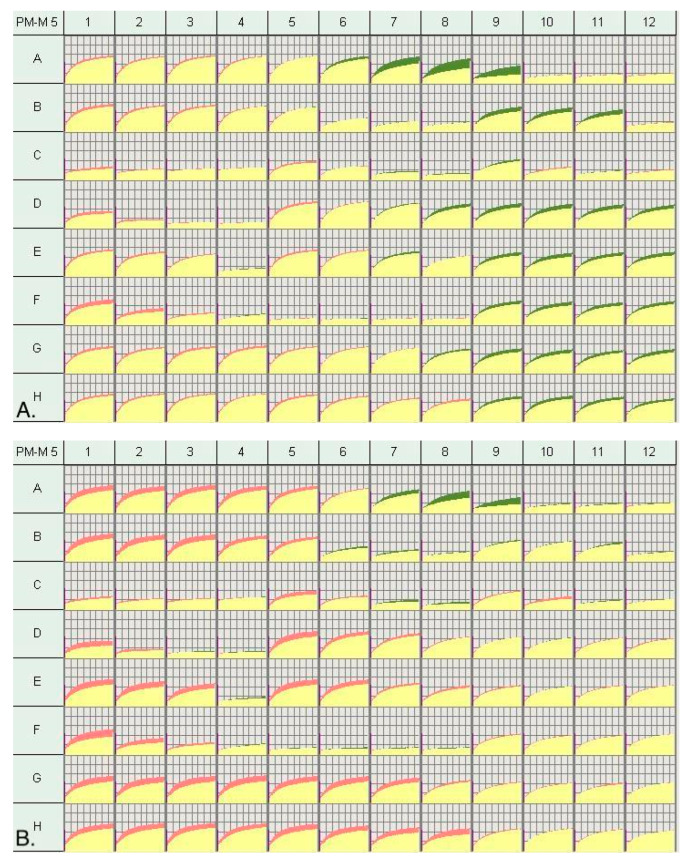

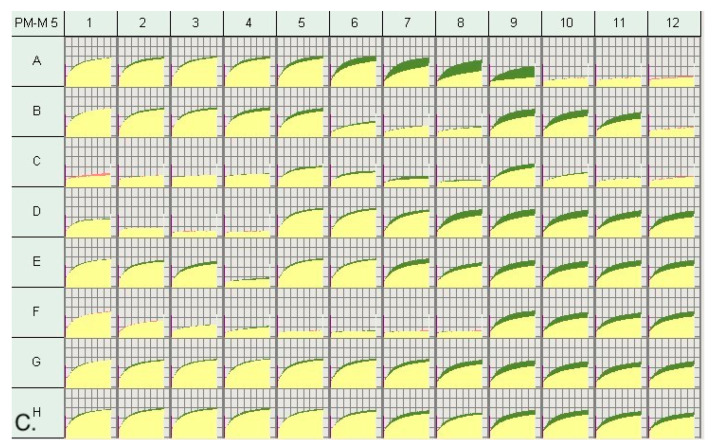
(**A**). Metabolic profile of 48 individuals with PMS vs. 50 controls. Red represents the wells in the control group that were higher and green represents the wells in the PMS cohort that were higher on the Biolog plate PM–M5. (**B**). Metabolic profile of 50 control individuals vs. 12 hGH low responders with PMS for plate PM-M5. (**C**). Metabolic Profile of 50 control individuals vs. 12 hGH high responders for plate PM–M5. Wells of interest were: NaCl (A5 to A9), Potassium Chloride (B9 to B11), Iodine (D9 to D12), Sodium Molybdate (E9 to E12), Sodium Nitrite (G9 to G12), and Magnesium Chloride (H9 to H12).

The results from the PM–M1 plate highlight clear differences in the utilization of energy sources between PMS and control LCLs. The PMS cohort showed an increased response to carboxylic acids (a-keto-butyric acid, or hexanoic acid) suggesting that alternative pathways for the production of cellular energy are being used to compensate for a lower ability to utilize main energy sources through the aerobic pathways (Table 1). The decrease in the utilization of main energy sources was further confirmed in PM–M2 and M3 plates, which showed lower levels of NADH detected in various wells that contained amino acids and dipeptides throughout the two plates. There was significantly increased production of NADH levels throughout the PM–M5 (Figure 1), which contained ionic species in groups of four wells with increasing dosage. The wells containing iodine, magnesium chloride, potassium chloride, sodium chloride, sodium molybdate, and sodium nitrite all showed increased levels of NADH (Table 1). Results from the PM–M6 plate showed increased NADH production in wells containing the highest doses of norepinephrine. However, all wells containing epinephrine and four out of the six wells containing dexamethasone and L-Leucine showed a reduction in NADH production. The response to metabolic stimulants such as triiodothyronine (T3) and 3-isobutyl-1-methylxanthine resulted in diminished NADH production. The low response to T3, a thyroid hormone, and the high response to iodine on PM–M5 suggests a potential disruption within the thyroid metabolic pathway. For plate PM–M7 there was a dose effect on glucose metabolism, showing increased NADH production for the highest concentrations of insulin, glucagon, and leptin within the wells (Table 1). Interestingly, neither the wells containing hGH nor the ones with IGF-1 resulted in significantly different PMS cells as compared to controls, suggesting these compounds may not have the same effects on the collective PMS population.

### 3.2. Metabolic Profile of High and Low Responders

**Figure 2:**
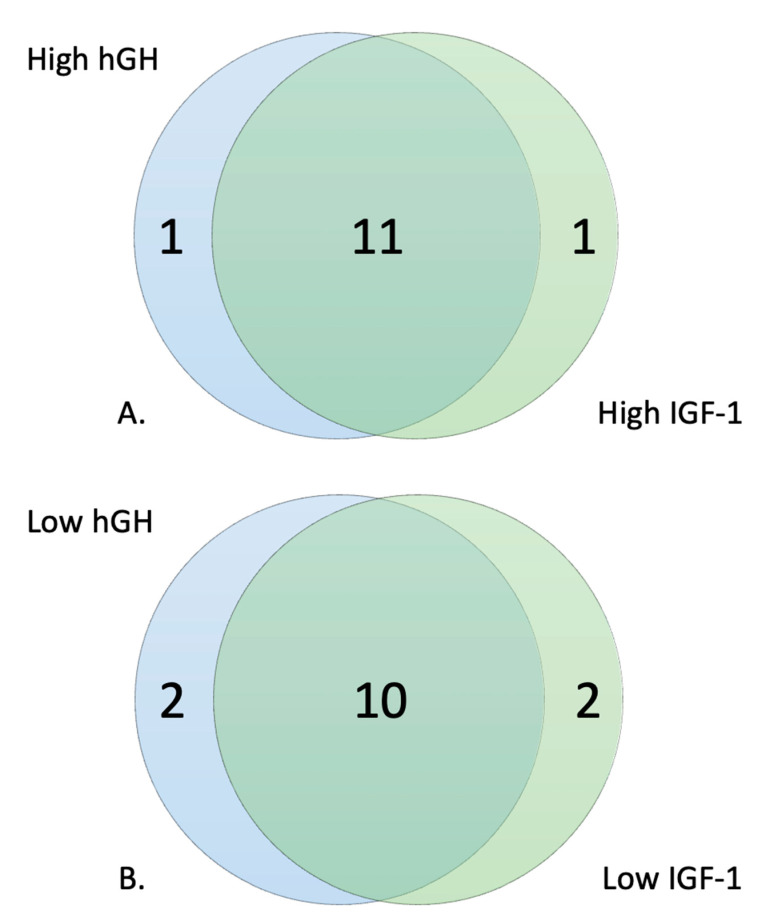
(**A**) Similarities between the selection of cell lines based on their metabolic response to hGH and IGF-1. (**A**) High responding cell lines and (**B**) low responding cell lines.

**Figure 3:**
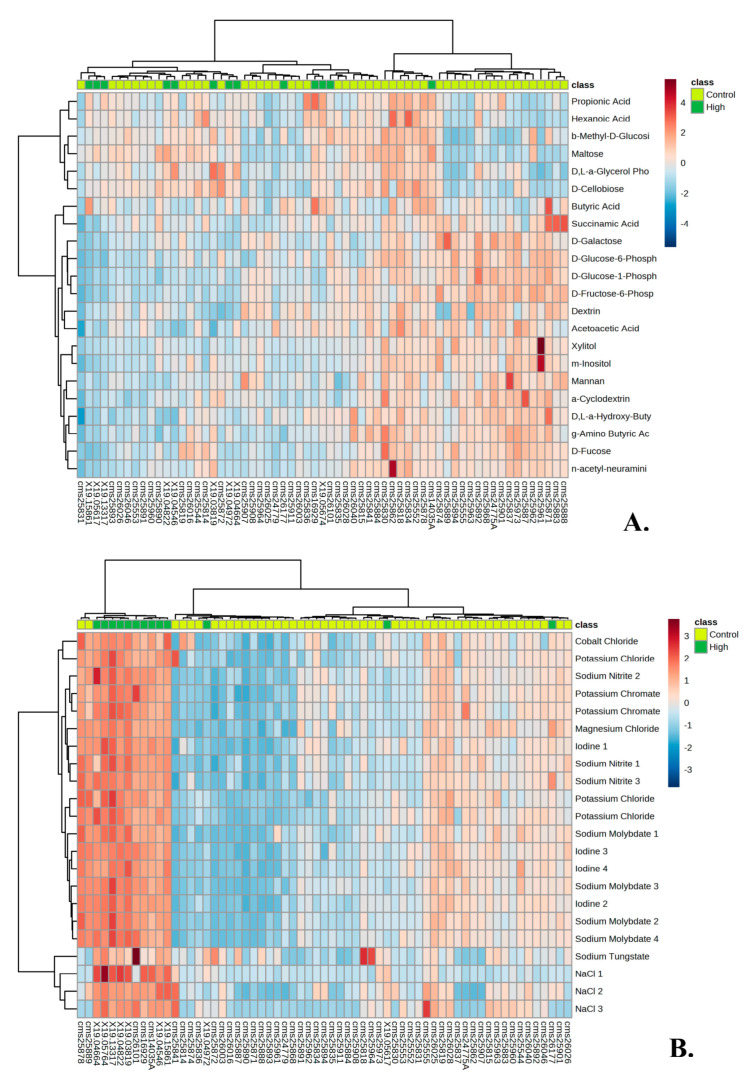
(**A**) Heat map of high responders and 50 control individuals’ metabolic response to the top 20 significant wells of plate PM–M1(energy sources). (**B**) Heat map of high responders and 50 control individuals’ metabolic response to the top 20 significant wells of plate PM–M5 (ionic species).

There was a large overlap between those who were high responders to IGF-1 and high responders to hGH, with 11 out of the 12 being the same individuals (Figure 2A). Heat maps of the top 20 significant wells for PM–M1 and PM–M5 showed the metabolic difference between high responders and the 50 control individuals (Figure 3A,B). It is interesting to notice how the high-responders (green in Figure 3A) are scattered in the heat map of PM–M1 (containing energy sources) but tightly clustered in the PM–M5 one, suggesting that the metabolic profile generated in response to ionic species may be a more reliable biomarker than the one detected in the presence of energy sources to select the high-responder subgroup in the PMS population. Analysis of the data from PM–M1 to M8 and TRP plates detected 290/776 (37.37%) significant wells in the high response hGH cohort and 293/776 (37.76%) in the high response IGF-1 cohort (Table 2) when compared to the control cohort. The high hGH responder cohort had eight wells that generated significantly lower levels of NADH than controls and the high IGF-1 responders had 10 wells significantly lower than controls. Between the two responder groups, 284 wells were significant for both high responder groups (Table 2).

**Figure 4:**
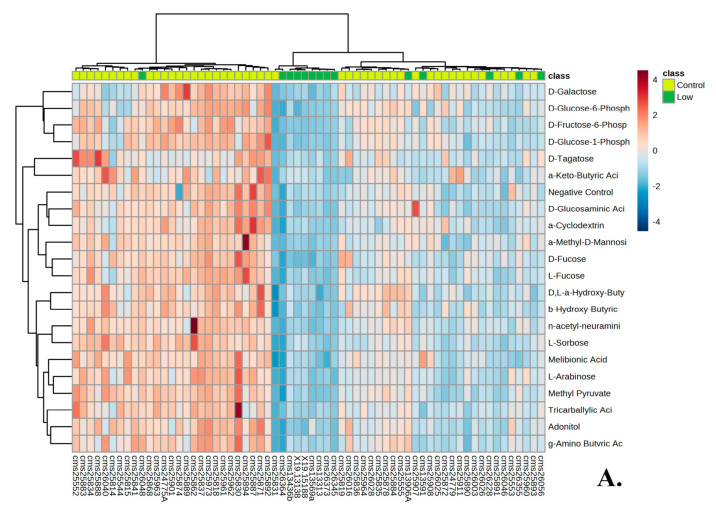

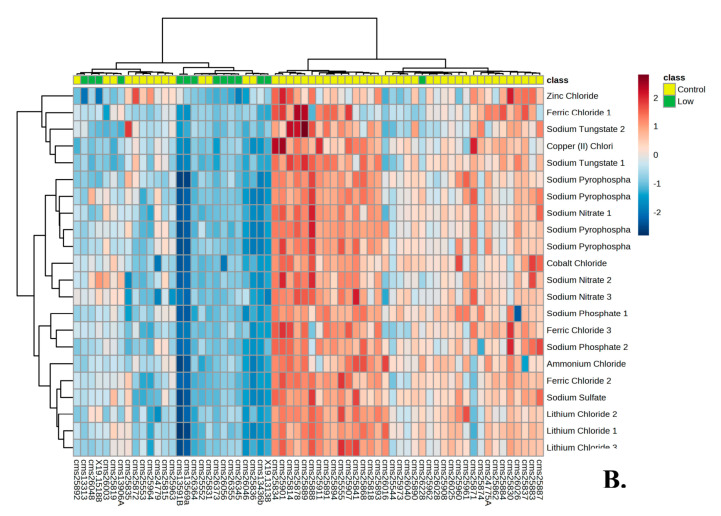
(**A**) Heat map of low responders and 50 control individuals’ metabolic response to the top 20 significant wells of plate PM–M1(energy sources). (**B**) Heat map of low responders and 50 control individuals’ metabolic response to the top 20 significant wells of plate PM–M5 (ionic species).

The selection of the low responders of hGH and IGF-1 resulted in 10 out of 12 individuals being the same between the two groups (Figure 2B). Heat maps of the top 20 significant wells for PM–M1 and PM–M5 showed the metabolic difference between low responders and the 50 control individuals (Figure 4A,B). Unlike the trend reported for the high responders, the low-responder sample (green in Figure 4A,B) clustered relatively tightly both in PM–M1 (energy sources) and PM–M5 (ions), indicating that this subgroup presents a highly recognizable metabolic profile in multiple assays and is thus more easily identifiable than the high responder subgroup within the PMS population. The low responders for hGH showed 514/776 significant wells (66.24%) across all PM–M and TRP plates and the low responders for IGF-1 showed 507/776 significant wells (65.33%). The low hGH and low IGF-1 responder cohorts only had one well each which generated an increased production of NADH compared to controls. The rest of the wells showed a reduction in the amount of NADH produced, as compared to controls. Between the two groups of low responders, 489 wells were significant for both low responder groups (Table 2). We are aware that the concentration range in the wells may span beyond the physiological values for these compounds. However, when we looked at the level of NADH production in all 12 wells, there were no outliers.

### 3.3. Response to hGH and IGF-1 in Comparison to Baseline Glucose Metabolism

**Figure 5:**
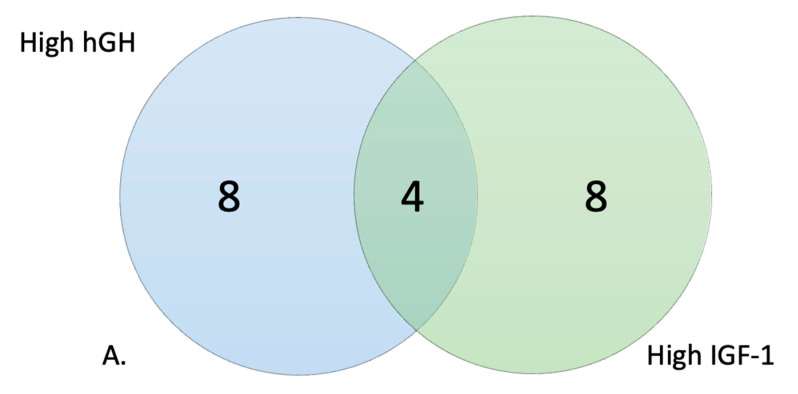

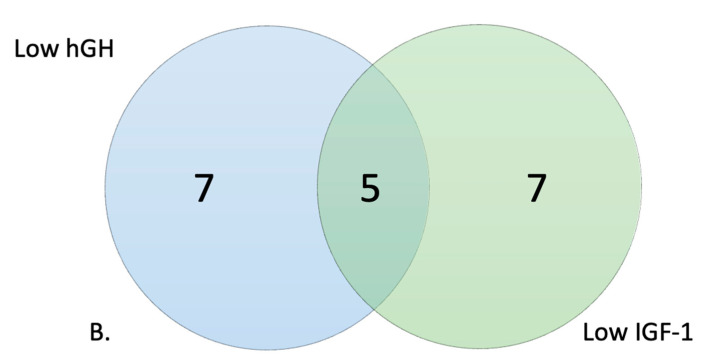
Similarities between the selection of cell lines based on their metabolic response to hGH and IGF-1 correlated to baseline glucose metabolism. (**A**) High responding cell lines and (**B**) low responding cell lines.

Since both hGH and IGF-1 have a direct influence on glucose metabolism, mimicking the anabolic effect of insulin, and because hypoglycemia is a potential side effect in individuals treated with IGF-1, we correlated the metabolic response to these two compounds to the baseline glucose metabolism. In the PM–M7 plate, the cells utilize glucose from the media to produce NADH in the presence of various metabolic effectors, including hGH (wells E1-E6) and IGF-1 (wells E7-E12), except for the first six wells (A1-A6), where no metabolic effector is present. We used these first six wells for each individual as controls, representing the baseline energetic metabolism of glucose. The data collected from the wells containing varying concentrations of hGH and IGF-1 were thus divided by the average of the six control wells, allowing us to observe how these compounds alter the baseline glucose metabolism in individuals with PMS. After adjusting for baseline glucose metabolism, the overlap between groups was lower, with the high responders having only 4 out of 12 individuals being the same between hGH and IGF-1, while 11 out of 12 were the same before correlation (Figure 5A). Similarly, the low responders had 7 out of 12 individuals that were the same as compared to the 10 out of 12 before the correlation (Figure 5B). The high responders had fewer overlap groups and were more different in terms of the cell lines selected for the top 25% based on the response to hGH and IGF-1. The low responders maintained a very sensitive overlap across hGH and IGF-1, having only three different cell lines from the initial group of 12 low responders. These results suggest that the primary metabolic effect exerted on the metabolic profile of PMS cells is not strictly connected to their influence on glucose metabolism.

## 4. Discussion

The metabolic profile assessed from LCLs of 48 individuals with PMS indicates an abnormal NADH production in the presence of major energy sources and a possible compensatory mechanism utilizing alternative energy sources within this population. The whole metabolic profile of the PMS population points towards the altered capacity of these cells to respond to external stimulation and challenging metabolic environments. Results from PM–M5 suggest that certain salts might induce an increased NADH production for PMS and therefore make these cells more sensitive to certain ionic species and/or pH levels. PMS LCLs also showed an abnormal response to certain metabolic stimulants and hormones regulating glucose metabolism. The reduced response to tryptophan was observed across the entire PMS cohort and remained present when the high and low responder groups were categorized is consistent with similar trends observed in patients with both isolated and syndromic ASD [26,27].

When stratifying the PMS population into low and high responders based on their response to hGH or IGF-1, the trend of lower production of NADH was not observed within the high responders of hGH and IGF-1, except for that of plate PM–M1 (Figure 3). The low-responder groups tended to respond poorly across all PM–M plates and lost the compensatory mechanism that was present in the metabolic profile of the complete PMS cohort (Figure 4). These trends can be seen within the heat maps, where the high responder group was high all across PM–M5 and generally low in the plates containing energy sources, such as PM–M1 (Figure 4). The heat maps focused on the low responder group continue to show the trend of this group responding low across all plates. The selection of high and low responders for either hGH or IGF-1 resulted in the same candidates being selected and having the same metabolic profiles across all PM–M plates (Figure 2). The number of wells that were significant and the same between hGH and IGF-1 for each group suggests that these compounds largely interact with the same pathways, validating the hypothesis that hGH may exert the same molecular and clinical effects as IGF-1 [21]. This selection based on metabolic response determined two clearly different metabolic endophenotypes based on the response to hGH and IGF-1. It is noteworthy that the whole PMS cohort did not show significant responses to either IGF-1 or hGH, supporting the preclinical stratification of the patients to identify the ones that might manifest the best or worst responses to the exposure to these compounds.

When stratifying the PMS population in correlation to the baseline glucose metabolism, the low responders to either hGH or IGF-1 remained similarly grouped, whereas the high responder group was very different. This could point towards another pathway that is not shared by hGH and IGF-1 being the cause of the metabolic effect. Assuming that hGH has a less relevant influence on the glucose metabolism pathway than IGF-1, these results support the utilization of hGH in clinical trials in place of IGF-1 to reduce the risk of hypoglycemia. Therefore, this secondary stratification of the PMS population could be another way to evaluate side effects linked to glucose metabolism and further classify individuals with PMS into subpopulations for clinical trials.

The proposed approach of stratification of a targeted cohort using metabolic profiling can be applied to other genetic conditions characterized by clinical variability, such as ASD. PMS was listed as a syndromic form of ASD in the DSM-IV-TR and autism or autistic traits are frequently reported in individuals with PMS [1,5,7]. Moreover, the beneficial effects reported in clinical trials using IGF-1 in PMS seem to target primarily the behavioral phenotype [2,19]. For these reasons, we suggest that metabolic profiling based on the response to IGF-1, hGH, or other biomarkers can significantly contribute to the stratification of cohorts of individuals with ASD, especially if clinically heterogeneous, and eventually to the identification and characterization of ASD endophenotypes, leading to a better diagnosis and management of the individuals affected with this disorder.

## 5. Conclusions

The results of this study indicate disruptions in the pathways involved in the utilization of major energy sources in LCLs from individuals with PMS. The PMS population can be stratified into subpopulations based on their metabolic response to hGH and IGF-1. The PM–M platform may represent an in vitro model to assess metabolic biomarkers to screen these subpopulations. This study based on the stratification of the PMS population has the potential to allow for better patient selection in clinical trials, identifying candidates that are metabolically predisposed to optimal responses to hGH and/or IGF-1 and minimal or no side effects. Future studies should focus on the correlation of metabolic endophenotypes to clinical symptoms and the use of biomarkers to select potential candidates for trial or identify how individuals are responding to the drug while in a clinical trial. Lastly, to validate our preliminary findings, we plan to conduct in vitro studies to observe if exposure to hGH or IGF-1 can correct the metabolic abnormalities observed in the high- and low-responder subpopulations.

## Figures and Tables

**Table 1:** Significant (BH corrected *p* < 0.05) wells in Biolog plates in terms of metabolic activity and their energy production in individuals with PMS when compared to a cohort of controls. Red downward arrows show reduced NADH production and green upward arrows show an upregulation in production. NADH production is an indicator of energy production.

Differential Compounds	Difference in NADH between Individuals with PMS and Controls (*p*-Values)	Differential Compounds	Difference in NADH between Individuals with PMS and Controls (*p*-Values)
**PM–M1:**			
B2: D-Glucose-1-Phosphate	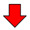 1 × 10^−5^	A3: Negative Control	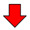 0.00876
D6: D-Fructose-6-Phosphate	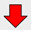 1 × 10^−5^	A1: Negative Control	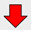 0.0094
B1: D-Glucose-6-Phosphate	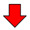 0.00007	A9: Maltose	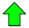 0.0094
E3: D-Galactose	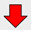 0.00013	F12: Citric Acid	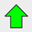 0.01144
D5: D-Fucose	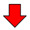 0.00015	E4: a-Methyl-D-Galactoside	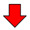 0.0116
C1: D-Glucosaminic Acid	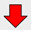 0.00061	H1: Acetoacetic Acid	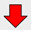 0.01346
H4: D,L-a-Hydroxy-Butyric Acid	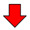 0.00102	C3: Chondroitin Sulfate	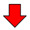 0.01663
H5: b-Hydroxy-Butyric Acid	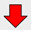 0.00232	E5: b-Methyl-D-Galactoside	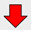 0.01724
H10: Propionic Acid	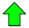 0.00265	A2: Negative Control	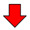 0.01906
A5: Dextrin	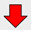 0.0029	A11: D-Cellobiose	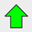 0.0205
E6: n-acetyl-neuraminic acid	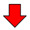 0.00464	B3: L-Glucose	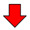 0.0205
B4: D-(+)-Glucose	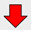 0.0061	D3: L-Rhamnose	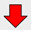 0.02218
B5: D-(+)-Glucose	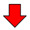 0.0061	C12: Turanose	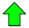 0.02388
C6: a-Methyl-D-Mannoside	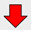 0.0061	D2: L-Sorbose	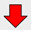 0.02388
E1: Melibionic Acid	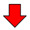 0.0061	D1: D-Tagatose	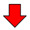 0.02581
F1: Adonitol	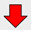 0.0061	E11: Adenosine	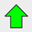 0.02603
F6: m-Inositol	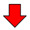 0.0061	E12: Inosine	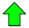 0.02603
G5: Pyruvic Acid	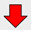 0.0061	F2: L-Arabinose	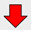 0.02603
H2: g-Amino Butyric Acid	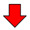 0.0061	F4: b-Methyl-D-Xyloside	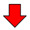 0.02603
A4: a-Cyclodextrin	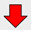 0.00676	G8: Succinic Acid	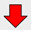 0.02603
C4: Mannan	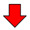 0.00676	H7: Butyric Acid	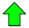 0.0276
C5: D-Mannose	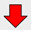 0.00676	B10: Salicin	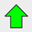 0.03039
D4: L-Fucose	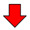 0.00676	B11: D-Sorbitol	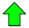 0.03039
F5: Xylitol	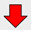 0.00676	G1: Tricarballylic Acid	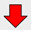 0.03039
G7: Succinamic Acid	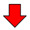 0.00676	E2: D-Melibiose	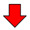 0.03823
H3: a-Keto-Butyric Acid	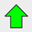 0.00676	G4: Methyl Pyruvate	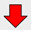 0.03823
H12: Hexanoic Acid	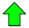 0.00717	A6: Glycogen	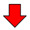 0.04949
			
**PM–M2:**			
E1: Ala-Pro	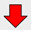 0.00446	C8: D-Threonine	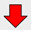 0.02213
F1: Arg-Leu	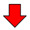 0.00446	D6: Ala-Gly	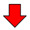 0.02213
H4: Glu-Val	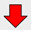 0.00446	F2: Arg-Lys	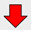 0.02213
D1: Ala-Arg	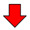 0.00491	F11: Asp-Ala	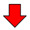 0.02213
B1: L-Aspartic Acid	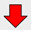 0.00708	G9: Glu-Ala	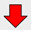 0.02213
E7: Arg-Ala	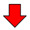 0.00708	H1: Glu-Ser	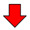 0.02213
F4: Arg-Phe	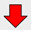 0.00708	F7: Arg-Tyr	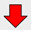 0.02334
D9: Ala-Leu	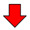 0.00735	G5: Asp-Lys	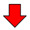 0.02847
G1: Asp-Glu	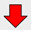 0.00735	D4: Ala-Glu	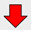 0.03188
H2: Glu-Trp	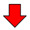 0.00862	E6: Ala-Val	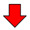 0.03495
D7: Ala-His	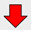 0.01228	C6: D-Serine	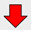 0.04029
E4: Ala-Trp	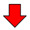 0.0135	H8: Gly-Ala	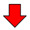 0.04029
E3: Ala-Thr	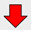 0.01606	D12: Ala-Phe	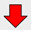 0.04078
G8: Asp-Val	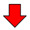 0.01606	A1: Negative Control	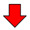 0.04624
D5: Ala-Gln	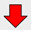 0.01718	A8: L-Alaninamide	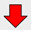 0.04624
F5: Arg-Ser	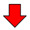 0.01718	B3: L-Glutamic Acid	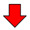 0.04624
G4: Asp-Leu	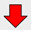 0.01744	D10: Ala-Lys	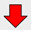 0.04624
F6: Arg-Trp	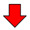 0.01814	G11: Glu-Glu	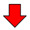 0.04624
A3: Negative Control	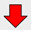 0.02156	F3: Arg-Met	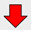 0.04674
D8: Ala-Ile	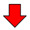 0.02156	G2: Asp-Gln	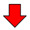 0.04674
G6: Asp-Phe	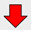 0.02156	A2: Negative Control	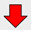 0.04747
G7: Asp-Trp	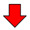 0.02207		
			
**PM–M3:**			
B1: Gly-Thr	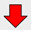 0.01047	E7: Leu-Phe	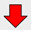 0.04043
C4: His-Tyr	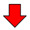 0.01228	F1: Lys-Ala	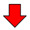 0.04043
D1: Ile-Leu	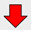 0.0157	F10: Lys-Phe	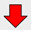 0.04043
A8: Gly-Lys	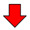 0.02377	C3: His-Trp	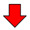 0.0411
C6: Ile-Ala	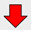 0.02377	D9: Leu-Ala	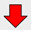 0.0411
C1: His-Pro	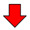 0.04043	E8: Leu-Pro	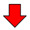 0.0411
D4: Ile-Pro	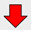 0.04043	A10: Gly-Phe	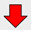 0.04198
D6: Ile-Trp	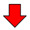 0.04043	E6: Leu-Met	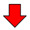 0.04583
E5: Leu-Leu	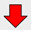 0.04043	D5: Ile-Ser	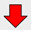 0.04805
			
**PM–M5:**			
A9: NaCl	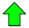 1 × 10^−5^	G9: Sodium Nitrite	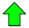 0.00196
A8: NaCl	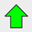 0.00001	E11: Sodium Molybdate	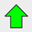 0.00286
A7: NaCl	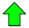 0.00002	F12: Potassium Chromate	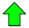 0.00366
B11: Potassium Chloride	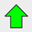 0.00018	H10: Magnesium Chloride	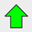 0.00366
F4: Sodium Tungstate	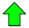 0.00018	F9: Potassium Chromate	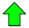 0.00623
F2: Sodium Tungstate	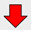 0.00052	G11: Sodium Nitrite	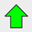 0.00644
D10: Iodine	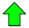 0.00074	H11: Magnesium Chloride	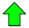 0.00974
B10: Potassium Chloride	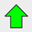 0.00077	F8: Sodium Orthovanadate	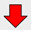 0.0103
D9: Iodine	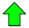 0.00089	G1: Sodium Pyrophosphate	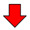 0.01031
B9: Potassium Chloride	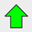 0.00111	H9: Magnesium Chloride	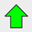 0.01031
E10: Sodium Molybdate	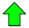 0.00111	C8: Manganese Chloride	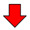 0.01134
E12: Sodium Molybdate	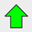 0.00111	D8: Cobalt Chloride	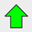 0.01134
F1: Sodium Tungstate	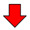 0.00111	D1: Copper (II) Chloride	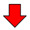 0.01536
F11: Potassium Chromate	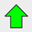 0.00123	G3: Sodium Pyrophosphate	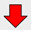 0.017
G10: Sodium Nitrite	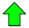 0.00123	H12: Magnesium Chloride	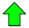 0.02089
G12: Sodium Nitrite	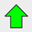 0.00123	D5: Cobalt Chloride	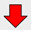 0.03199
F10: Potassium Chromate	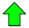 0.0014	D4: Copper (II) Chloride	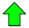 0.0427
D12: Iodine	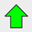 0.00161	G4: Sodium Pyrophosphate	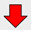 0.0458
E9: Sodium Molybdate	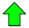 0.00161	C12: Zinc Chloride	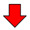 0.04771
D11: Iodine	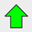 0.00171		
			
**PM–M6:**			
A12: Dibutyryl-cAMP	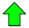 0.01099	D2: L-Leucine	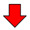 0.02157
B1: 3-Isobutyl-1-Methylxanthine	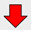 0.01099	D4: L-Leucine	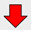 0.02157
B2: 3-Isobutyl-1-Methylxanthine	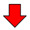 0.01099	F2: Dexamethasone	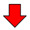 0.02253
B4: 3-Isobutyl-1-Methylxanthine	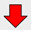 0.01099	C12: Norepinephrine	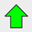 0.02864
C1: Epinephrine	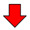 0.01099	E4: Triiodothyronine	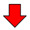 0.02864
D1: L-Leucine	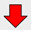 0.01099	F3: Dexamethasone	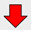 0.0306
E1: Triiodothyronine	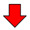 0.01099	D12: Creatine	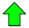 0.03105
F1: Dexamethasone	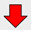 0.01099	C2: Epinephrine	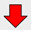 0.03287
A11: Dibutyryl-cAMP	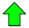 0.01103	C4: Epinephrine	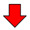 0.03376
B3: 3-Isobutyl-1-Methylxanthine	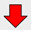 0.01103	A1: Negative Control	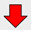 0.03388
B10: Caffeine	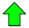 0.01537	A9: Dibutyryl-cAMP	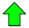 0.03388
E3: Triiodothyronine	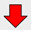 0.01537	C11: Norepinephrine	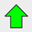 0.03388
A10: Dibutyryl-cAMP	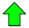 0.02017	A2: Negative Control	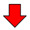 0.03607
B11: Caffeine	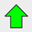 0.02017	B9: Caffeine	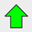 0.03607
D3: L-Leucine	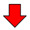 0.02017	C5: Epinephrine	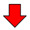 0.03607
F4: Dexamethasone	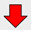 0.02017	F12: Hydrocortisone	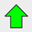 0.03607
G1: Progesterone	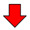 0.02017	A2: Negative Control	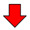 0.03652
B6: 3-Isobutyl-1-Methylxanthine	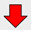 0.02157	G5: Progesterone	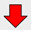 0.03748
C3: Epinephrine	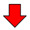 0.02157	H9: Aldosterone	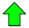 0.03748
C6: Epinephrine	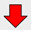 0.02157	F6: Dexamethasone	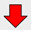 0.03951
			
**PM–M7:**			
A12: Insulin	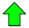 0.01964	A10: Insulin	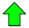 0.02473
B11: Glucagon	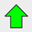 0.02006	B12: Glucagon	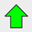 0.02765
A11: Insulin	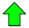 0.02088	C10: Leptin	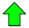 0.03238
B10: Glucagon	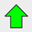 0.02088	C11: Leptin	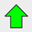 0.03238
B9: Glucagon	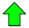 0.02437	C12: Leptin	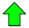 0.03238
			
**TRP:**			
A1: a-D-Glucose	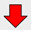 1 × 10^−5^	E1: Trp-Lys	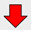 0.00023
D1: Trp-Gly	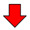 0.00003	F1: Trp-Ala	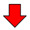 0.00067
C1: L-Tryptophan	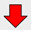 0.00005	H1: Trp-Leu	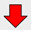 0.00357

**Table 2:** Number of significant wells (*p* < 0.05) on each Biolog plate (PM–M1 to M8, and TRP) for the stratified groups of low and high responders to hGH and IGF-1 when compared to the control cohort. The number of wells that were significant and the number of wells in common between the high responders to hGH and IGF-1, and between the low responders to hGH and IGF-1 show the similarity between responders in relation to these two compounds.

	**hGH High Responders**	**%**	**IGF-1 High Responders**	**%**	**# Wells in Common**
**PM–M1:**	3	3.13%	3	3.13%	3
**PM–M2:**	0	0.00%	0	0.00%	0
**PM–M3:**	0	0.00%	0	0.00%	0
**PM–M4:**	0	0.00%	0	0.00%	0
**PM–M5:**	39	40.63%	43	44.79%	39
**PM–M6:**	63	65.63%	62	64.58%	59
**PM–M7:**	93	96.88%	91	94.79%	91
**PM–M8:**	89	92.71%	89	92.71%	89
**TRP:**	3	37.50%	5	62.50%	3
	**hGH Low responders**	**%**	**IGF-1 Low responders**	**%**	**# Wells in common**
**PM–M1:**	52	54.17%	49	51.04%	48
**PM–M2:**	60	62.50%	66	68.75%	55
**PM–M3:**	71	73.96%	62	64.58%	60
**PM–M4:**	0	0.00%	0	0.00%	0
**PM–M5:**	50	52.08%	49	51.04%	48
**PM–M6:**	85	88.54%	88	91.67%	85
**PM–M7:**	95	98.96%	95	98.96%	95
**PM–M8:**	96	100.00%	96	100.00%	96
**TRP:**	5	62.50%	2	25.00%	2

## Data Availability

All data generated or analyzed during this study are included in this published article and are available by request.
